# Aneuploidy enables adaptation to brefeldin A in *Candida albicans*

**DOI:** 10.3389/fcimb.2025.1562726

**Published:** 2025-04-28

**Authors:** Weifang Wang, Chen Wang, Yubo Dong, Feng Yang, Yi Xu

**Affiliations:** ^1^ Jinzhou Medical University Graduate Training Base (The 960th Hospital of PLA), Jinan, China; ^2^ Department of Pharmacy, The 960th Hospital of PLA, Jinan, China; ^3^ Department of Pharmacology, Shanghai Tenth People’s Hospital, Tongji University School of Medicine, Shanghai, China

**Keywords:** *Candida albicans*, aneuploidy, ER stress, brefeldin A, genome instability

## Abstract

*Candida albicans* is the most prevalent opportunistic fungal pathogen. Both *in vivo* and *in vitro* studies have demonstrated that genome plasticity is a hallmark of *C. albicans*. While aneuploidy formation is a well-documented adaptive mechanism under various stress conditions, the response to brefeldin A—a compound that induces endoplasmic reticulum stress—remains poorly understood. In this study, we demonstrate that *C. albicans* adapts to subinhibitory and inhibitory concentrations of brefeldin A, primarily through the formation of chromosome 3 trisomy. These aneuploid strains were found to be unstable, reverting to euploidy in the absence of stress, accompanied by a loss of brefeldin A tolerance. We identified at least two genes on chromosome 3, *SEC7* and *CDR1*, that contribute to this adaptive response. Notably, higher concentrations of brefeldin A selected for strains with increasingly complex aneuploidies. Our findings underscore the remarkable genomic plasticity of *C. albicans* and reveal aneuploidy as a reversible mechanism for adapting to brefeldin A stress. This study provides new insights into the role of aneuploidy in fungal adaptation and offers potential implications for understanding drug resistance mechanisms in pathogenic fungi.

## Introduction

*Candida albicans* is a common member of the human microbiota, typically residing as a commensal organism in the oral cavity, gastrointestinal tract, and vagina. However, it is also the primary opportunistic fungal pathogen in humans, responsible for a wide range of infections. These include superficial conditions such as oral and vaginal candidiasis, as well as life-threatening systemic infections associated with high morbidity and mortality (Ciurea et al., 2020). In recent years, the population of immunocompromised individuals at risk for these infections has steadily increased, leading to a rise in opportunistic fungal infections. This trend has established these infections as a significant public health concern and a growing economic burden (Benedict et al., 2019). Among *Candida* species, *C. albicans* is the most commonly isolated pathogen in immunocompromised patients and remains the leading cause of candidiasis (Pfaller and Diekema, 2007).

The endoplasmic reticulum (ER) is a ubiquitous organelle in eukaryotic cells and serves as the primary site for protein folding and maturation. Given its central role in maintaining cellular homeostasis, the ER is highly sensitive to stress-induced changes in intracellular energy levels, oxidative status, and calcium ion concentrations. Consequently, various physiological and pathogenic factors can disrupt ER function, leading to the accumulation of unfolded or misfolded proteins within the ER lumen (Han and Kaufman, 2016; Wang and Kaufman, 2016). In response, cells activate the unfolded protein response (UPR), a conserved signaling pathway that restores ER protein-folding capacity to meet cellular demands (Bhattarai et al., 2021). However, cells also employ UPR-independent mechanisms to adapt to ER stress. For instance, in the haploid model organism *Saccharomyces cerevisiae*, aneuploidy of chromosome II confers tolerance to tunicamycin-induced ER stress independently of the UPR. This adaptation is mediated by at least three genes on chromosome II: *ALG7*, *PRE7*, and *YBR085C-A* (Beaupere et al., 2018). Similarly, in the diploid pathogen *Candida albicans*, trisomy of chromosome 2 enables rapid adaptation to both mild and severe ER stress induced by tunicamycin. At least four genes on chromosome 2—*ALG7*, *RTA2*, *RTA3*, and *MKK2*—have been implicated in this adaptive response (Yang et al., 2021a).

Brefeldin A (BFA) is a fungal fatty acid metabolite initially identified for its ability to inhibit viral replication. Subsequent studies revealed its role as a potent inhibitor of protein secretion, primarily by disrupting intracellular vesicular trafficking pathways. BFA exerts its effects by targeting ADP-ribosylation factor (Arf) proteins, which are key regulators of membrane traffic in eukaryotic cells. The activity of Arf proteins is modulated by guanine nucleotide-exchange factors (GEFs), which promote the exchange of GDP for GTP on Arf proteins via their catalytic Sec7 domain. BFA acts as an uncompetitive inhibitor by binding to the transient Arf–GDP–Arf GEF complex, thereby blocking nucleotide exchange catalyzed by the Sec7 domain (Robineau et al., 2000). This inhibition disrupts protein transport between the endoplasmic reticulum (ER) and the Golgi apparatus. As a result, BFA treatment leads to the accumulation of proteins in the ER, induction of ER stress, and ultimately, apoptosis.

In *Schizosaccharomyces pombe*, resistance to BFA has been linked to increased copy numbers of multidrug resistance transporter genes (Turi and Rose, 1995). In mammalian cells, overexpression of the GEF subfamily gene *GBF1* confers BFA resistance (Kawamoto et al., 2002). A recent study in *Cryptococcus neoformans* revealed that BFA resistance results from aneuploidy-mediated increases in the copy numbers of *SEC7* and the efflux gene *AFR1* (Zhang et al., 2024).

Although BFA is not a substrate of efflux pumps in human cells (Zahra et al., 2020), it functions as an inhibitor of both the ATP-binding cassette (ABC) superfamily and the major facilitator superfamily (MFS) efflux pumps in *C. albicans* (Diwischek et al., 2009). BFA exhibits antifungal activity against *C. albicans* (Hayashi et al., 1974) and shows synergistic effects with fluconazole—a widely used antifungal drug—against drug-resistant clinical isolates of *C. albicans* and non-*albicans Candida* species (Epp et al., 2010). Despite its potent activity, the mechanisms underlying BFA adaptation in *Candida* species remain poorly understood.

In this study, we demonstrated that the *C. albicans* reference strain SC5314 can adapt to both subinhibitory and inhibitory concentrations of BFA, primarily through the formation of chromosome 3 trisomy (Chr3x3). However, in the absence of BFA, these aneuploid strains were unstable and spontaneously reverted to euploidy, accompanied by a concomitant loss of BFA tolerance. We identified two genes on Chr3x3, *SEC7*, and *CDR1*, that contribute to this tolerance. Notably, prolonged exposure to increasing concentrations of BFA in Ch3x3-bearing strains led to the emergence of more complex karyotypes, suggesting that higher BFA levels select for adaptors with progressively intricate aneuploidies. Our findings underscore the remarkable genomic plasticity of *C. albicans* and establish aneuploidy as a key mechanism of adaptation to BFA in this pathogen.

## Materials and methods

### Strains and growth conditions

*C. albicans* lab strain SC5314 was used as the wild-type strain. Stock cultures were preserved in 25% glycerol and maintained at – 80°C. Cells were routinely grown in yeast extract–peptone–dextrose (YPD) medium (1% [w/v] yeast extract, 2% [w/v] peptone, and 2% [w/v] d-glucose) at 37°C in a shaking incubator at 150–200 rpm. For solid medium, 2% (w/v) agar was added. For the selection of gene knockout strains, YPD agar containing 400 μg/mL nourseothricin (Werner BioAgents, Jena, Thuringia, Germany) medium was used.

### Growth curves

Cells were suspended in YPD broth, and cell densities were adjusted to 2.5 × 10^3^ cells/mL in YPD broth with or without BFA in a 96-well plate. The plate was incubated at 37°C. OD_595_ was monitored in a Tecan plate reader (Infinite F200 PRO, Tecan, Switzerland, Männedorf, Zürich, Switzerland) at 15-min time intervals for 24 h. Data are represented as the mean ± SD of three biological replicates.

### Spot assay

Cells were suspended in distilled water and adjusted to 1 × 10^7^ cells/mL; 3 µL of 10-fold serial dilutions were spotted on YPD with or without BFA (control). Plates were incubated at 37°C and photographed after 2 days.

### Obtaining adaptors using a low amount of brefeldin A

Approximately 2.5 × 10^3^ cells/mL of SC5314 were inoculated into 1.5 mL of YPD broth containing 4 µg/mL of BFA. After 24 h of incubation with shaking, the culture was washed and diluted with distilled water, approximately 300 cells were spread on YPD plates and incubated at 37°C for 36 h. 120 colonies were randomly tested for tolerance to 32 μg/mL BFA. The plates were incubated at 37°C for 36 h and then photographed.

### Obtaining adaptors using a high amount of brefeldin A

Cells were suspended in distilled water, and the cell density was adjusted to 1 × 10^7^ cells/mL. A 100-µL aliquot of the cell suspension was placed on YPD plates supplemented with BFA (32 μg/mL for SC5314 and 128 μg/mL for No. 1). The plates were incubated at 37°C for 3 days. Eighteen adaptors were randomly chosen. The adaptors were streaked onto YPD plates and incubated at 37°C for 36 h. For each adaptor, four to six colonies of similar size were selected and frozen in 1 mL of 25% glycerol at − 80°C.

### Genome instability assay

Four adaptors with different karyotypes were daily passaged in YPD broth. After 10 passages, the cultures were washed and diluted with distilled water. Approximately 100 cells were spread on YPD plates. Four to six colonies of similar size were randomly selected and frozen in 25% glycerol.

### Gene deletions

Gene deletions were constructed using the *NAT1* flipper cassette as described previously (Yang et al., 2019). Approximately 500 bp of the upstream and downstream regions of the target gene were amplified using SC5314 genomic DNA as a template. The 3′ end of the upstream forward primers overlapped with the 5′ end of the *NAT1* flipper, and the 5′ end of the downstream reverse primers overlapped with the 3′ end of the *NAT1* flipper. The amplicon and plasmid pJK863 were used as templates to fuse the upstream region to the 5′ region of the cassette and the downstream region to the 3′ region of the cassette. The fusion products were transformed into *C. albicans* using the lithium acetate method (Wilson et al., 2000), and transformants were selected on YPD plates supplemented with 400 µg/mL of nourseothricin (NAT). Positive transformants were confirmed by diagnostic PCRs using primers that annealed outside the flanking homology regions. The NAT1 flipper was evicted by streaking the clones on YNB-BSA plates, which induces the Flp recombinase. All primer sequences are listed in **Supplementary Table S1**.

### Next-generation sequencing

DNA extraction, library construction, and sequencing were performed as described previously (Yang et al., 2021b). Data were visualized using Ymap (Abbey et al., 2014). Raw fastq files were uploaded to YMAP (version 1.0) (http://lovelace.cs.umn.edu/Ymap/). Read depth was plotted as a function of chromosome position using the Assembly 22 version of the SC5314 reference genome (http://www.candidagenome.org/download/sequence/C_albicans_SC5314/Assembly22/current/C_albicans_SC5314_A22_current_chromosomes.fasta.gz).

### Statistical analysis

Significance analysis of differences between growth curves was performed using a two-tailed paired *t*-test in GraphPad Prism (version 5.01).

## Results

### Antifungal activity of brefeldin A against *C. albicans*

**Figure 1:**
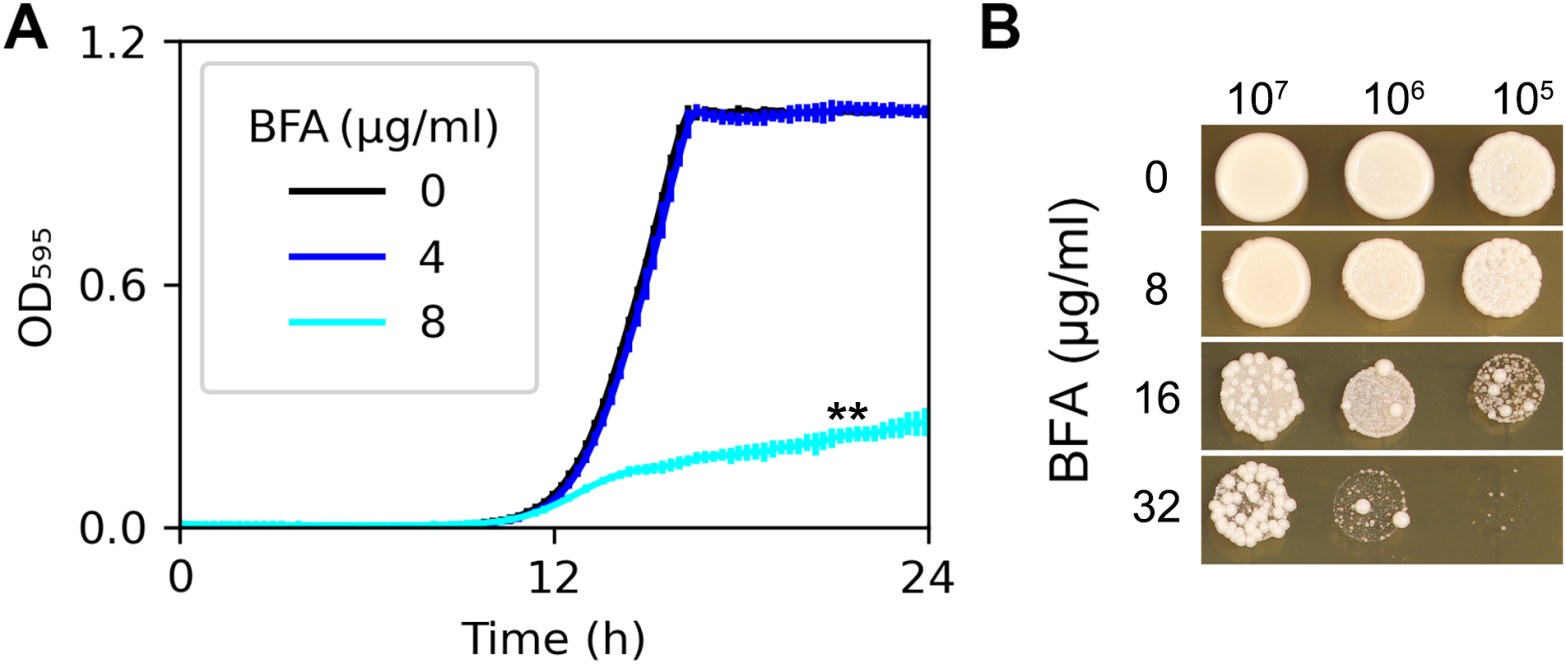
Susceptibility of SC5314 to brefeldin A. In growth curve assay **(A)**, approximately 2.5 × 10^3^ cells/mL of SC5314 were grown in 150 μL of YPD broth with or without BFA. Growth was monitored at 30°C using a plate reader (Infinite F200 PRO; Tecan, Switzerland) at 15-min time intervals for 24 (h) Data are represented as the mean ± SD of three biological replicates. In the spot assay **(B)**, cell densities were adjusted to 1 × 10^7^ cells/mL, and 3 µL of 10-fold serial dilutions were spotted onto YPD agar plates supplemented with BFA. Plates were incubated at 37°C for 48 h and then photographed. ^**^ *p*-values < 0.001, as determined by two-tailed Student’s *t*-test.

To evaluate the antifungal efficacy of BFA against *C. albicans*, two assays were conducted. In the growth curve assay (**Figure 1A**), the laboratory strain SC5314 was cultured in YPD broth supplemented with varying concentrations of BFA. While 4 μg/mL of BFA showed no significant growth inhibition compared to the YPD control, 8 μg/mL of BFA markedly reduced growth (*p* < 0.001, Student’s *t*-test). In contrast, the spot assay (**Figure 1B**) revealed that 8 μg/mL of BFA did not significantly inhibit growth on a plate. This discrepancy aligns with previous reports indicating that BFA exhibits stronger antifungal activity in liquid media compared to solid media (Xu et al., 2007).

### Exposure to a subinhibitory amount of brefeldin A selects aneuploid adaptors

**Figure 2:**
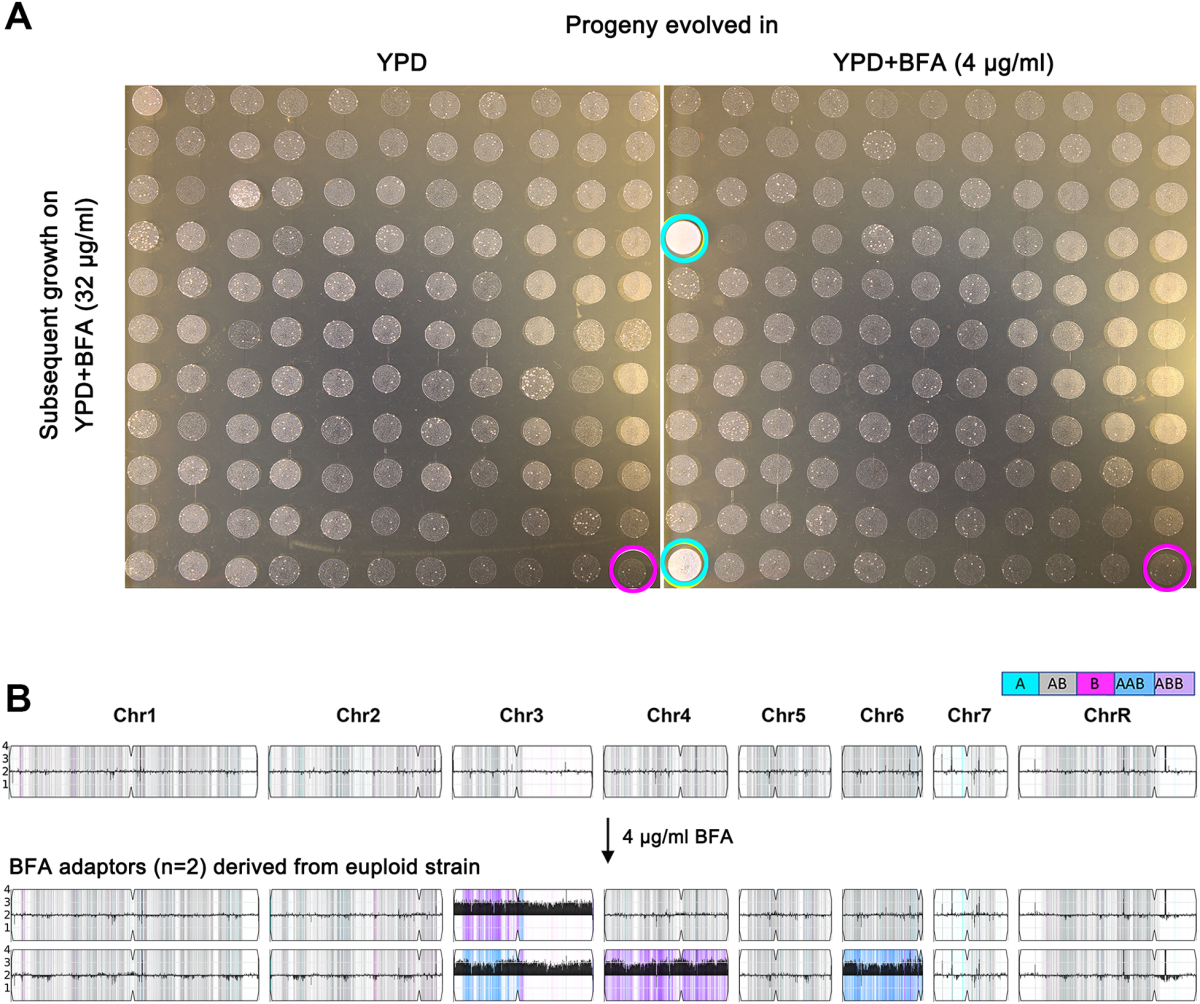
Exposure to a low amount of brefeldin A selects tolerant aneuploid adaptors. Approximately 2.5 × 10^3^ cells/mL of SC5314 were grown in 1.5 mL of YPD broth with or without 4 μg/mL of BFA. After 24 h of growth, the cultures were washed, diluted, and plated on YPD. A total of 120 colonies were randomly selected by spot assay on YPD plates supplemented with 32 μg/mL of BFA **(A)**. Magenta circles indicate the parent strain, and cyan circles indicate the tolerant adaptors. In **(B)**, the karyotypes of two BFA-tolerant adaptors were visualized using Ymap (Abbey et al., 2014). Read depth (normalized to that of the diploid parent) is shown on the *y*-axis on a log_2_ scale converted to absolute copy numbers (1–4). Allelic ratios (A:B) are color-coded as follows: gray, 1:1 (A/B); cyan, 1:0 (A or A/A); magenta, 0:1 (B or B/B); purple, 1:2 (A/B/B); and blue, 2:1 (A/A/B).

To determine whether short-term exposure to subinhibitory concentrations of BFA could select for BFA-tolerant adaptors, SC5314 was grown in YPD broth supplemented with 4 μg/mL of BFA for 24 h. The culture was then washed, diluted, and plated on YPD agar. A total of 120 randomly selected colonies were tested using a spot assay. None of the colonies evolved in YPD broth without BFA exhibited enhanced growth compared to the parent strain (red circle, **Figure 2A**, left panel). In contrast, among the colonies that evolved in YPD with BFA, two isolates (cyan circles, **Figure 2A**, right panel) demonstrated superior growth relative to the parent strain (white circle, **Figure 2A**, right panel). Whole-genome sequencing of these two tolerant adaptors revealed distinct aneuploidies: one adaptor exhibited trisomy of the B homolog of chromosome 3 (Chr3x3), while the other adaptor displayed trisomies of the A homologs of chromosomes 3 and 6, as well as the B homolog of chromosome 4 (Chrs(3,4,6)x3) (**Figure 2B**). These findings demonstrate that even short-term exposure to subinhibitory concentrations of BFA is sufficient to select aneuploid adaptors with enhanced BFA tolerance.

### Exposure to a high amount of brefeldin A selects aneuploid adaptors

**Figure 3:**
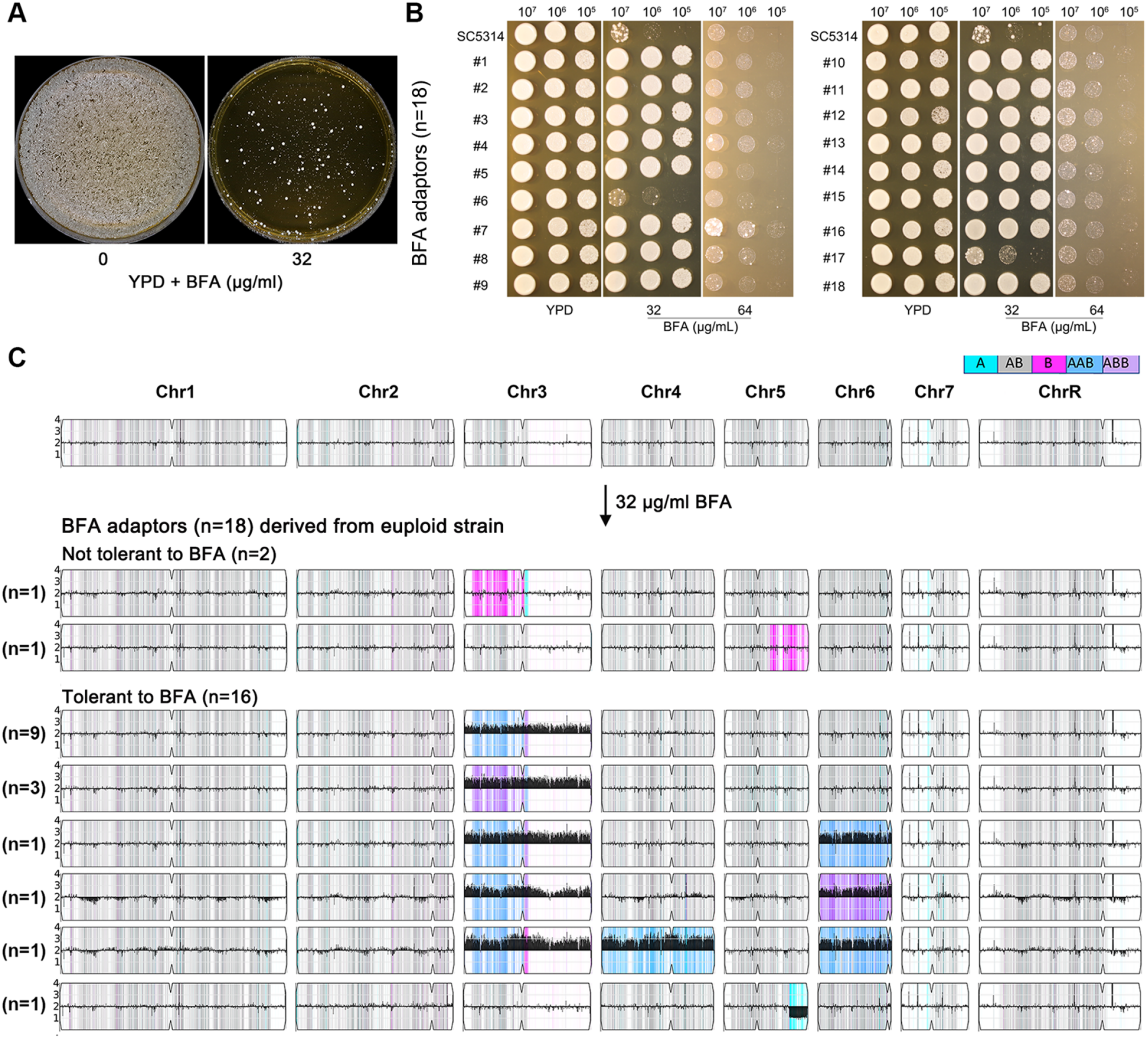
Aneuploidy enabled adaptation to a high amount of brefeldin A. In **(A)**, approximately 1 million cells of SC5314 were spread on a YPD plate or YPD plate supplemented with 32 μg/mL of BFA. The plates were incubated at 37°C for 3 days and then photographed. Eighteen adaptors (No. 1 to No. 18) were randomly selected and tested by spot assay on YPD plates supplemented with 32 and 64 μg/mL of BFA **(B)**. In **(C)**, karyotypes of all 18 adaptors were visualized using Ymap. The number of adaptors bearing the same karyotypes was indicated in the figure.

To investigate how *C. albicans* adapts to high concentrations of BFA, approximately 1 million SC5314 cells were plated on YPD agar supplemented with 32 μg/mL of BFA (**Figure 3A**). From these, 18 randomly selected adaptors (No. 1 to No. 18) were tested for BFA tolerance using a spot assay (**Figure 3B**). Two adaptors (No. 6 and No. 17) showed no increased tolerance compared to the parent strain, while the remaining 16 adaptors exhibited significantly enhanced BFA tolerance. Whole-genome sequencing of all 18 adaptors revealed distinct genomic changes. The two nontolerant adaptors were euploid but exhibited loss of heterozygosity (LOH) events: No. 6 had LOH on the left arm of chromosome 3, and No. 17 had LOH on a segment of the right arm of chromosome 5 (**Figure 3C**). In contrast, all 16 BFA-tolerant adaptors were aneuploid. Among these, nine adaptors carried trisomy of the A homolog of chromosome 3 (Chr3x3, AAB), three adaptors had trisomy of the B homolog of chromosome 3 (Chr3x3, ABB), and one adaptor displayed a segmental deletion on the right arm of the B homolog of chromosome 5 (from 0.92 Mb to the telomere). The remaining three adaptors exhibited complex aneuploidies involving at least two chromosomes: one adaptor had trisomies of the A homologs of chromosomes 3 and 6, another had trisomy of the A homolog of chromosome 3 and the B homolog of chromosome 6, and the third had trisomies of the A homologs of chromosomes 3, 4, and 6. These results demonstrate that all adaptors underwent genomic changes, with nontolerant adaptors showing LOH events and tolerant adaptors primarily exhibiting trisomy of chromosome 3, either alone or in combination with aneuploidies of other chromosomes.

### Genes on Chr3 associated with brefeldin A tolerance

**Figure 4:**
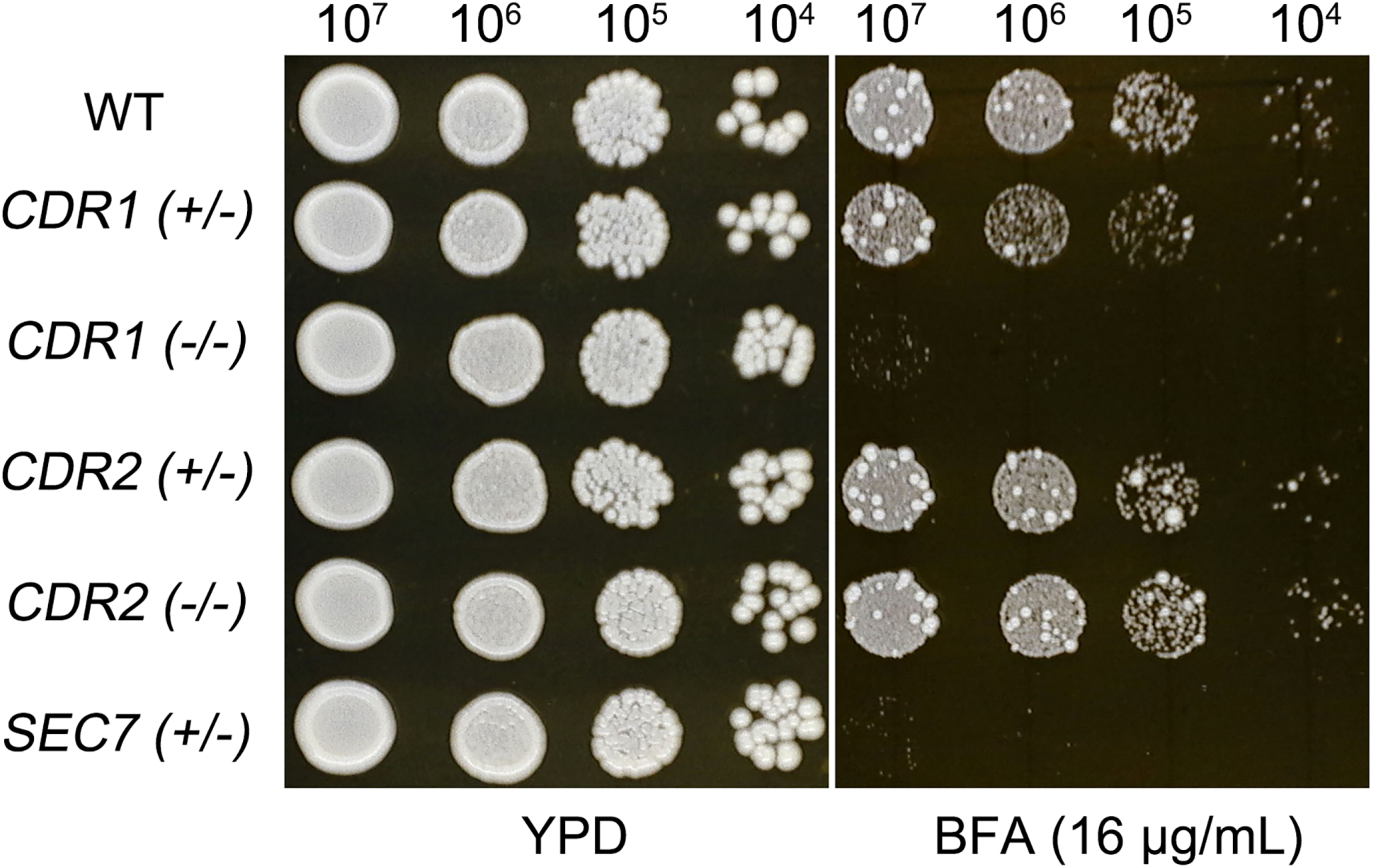
Chr3 genes associated with brefeldin A tolerance. Strains with heterozygous deletion of *SEC7* and both heterozygous and homozygous deletions of *CDR1* and *CDR*2 were assessed for BFA tolerance in comparison to the wild-type strain SC5314. Cells were spotted on TPD agar plates supplemented with BFA and incubated at 30°C for 48 h before being photographed.

In the *C. albicans* genome, *SEC7* is located on chromosome 3, as are *CDR1* and *CDR2*, which encode multidrug transporters of the ATP-binding cassette (ABC) superfamily. *SEC7* is an essential gene, and we found that the deletion of a single allele was sufficient to confer hypersensitivity to BFA. Deletion of one allele of *CDR1* caused slightly increased sensitivity to BFA, while deletion of both alleles resulted in hypersensitivity. However, deletion of one or both alleles of *CDR2* had no effect on BFA sensitivity (**Figure 4**). These results indicate that, on chromosome 3, *SEC7* and *CDR1* are associated with BFA tolerance, while *CDR2* does not play a significant role in this adaptive response.

### Genomic and phenotypic instability of aneuploids

**Figure 5:**
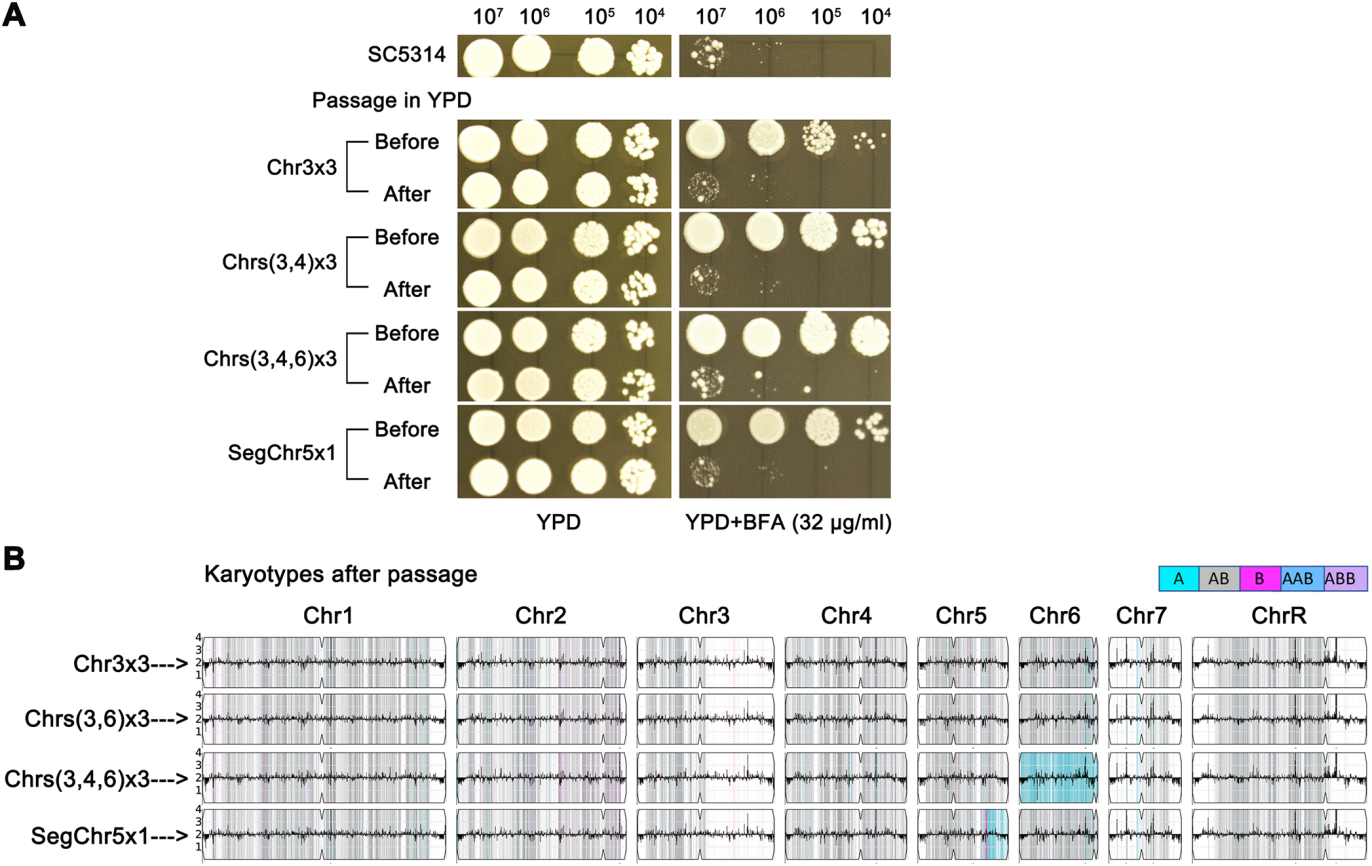
Instability of aneuploids. **(A)** Four aneuploid strains with distinct karyotypes were daily passaged in YPD broth. Tolerance to BFA before and after passaging was tested by spot assay. **(B)** After passage, the adaptors were sequenced. Karyotypes before passing were shown on the left panel.

In *C. albicans*, *C. auris*, and *C. neoformans*, specific aneuploidies confer tolerance to particular antifungal drugs, but these aneuploid strains are often unstable. In rich media under stress-free conditions, aneuploids spontaneously revert to euploidy, resulting in the loss of drug tolerance (Sionov et al., 2010; Yang et al., 2013; Yang et al., 2019; Bing et al., 2020; Chang et al., 2021). To determine whether aneuploid BFA adaptors exhibit similar instability, we tested four adaptors with distinct karyotypes: No. 1 (Chr3x3), No. 10 (Chrs(3,6)x3), No. 12 (Chrs(3,4,6)x3), and No. 16 (SegChr5x1). These strains were passaged daily in YPD broth for 10 generations and then assessed for BFA tolerance (**Figure 5A**). All four evolved progeny lost their BFA tolerance. Whole-genome sequencing revealed that each progeny had reverted to euploidy (**Figure 5B**). These findings demonstrate that aneuploid BFA adaptors are unstable, spontaneously reverting to euploidy in the absence of stress. This genomic instability directly correlates with the loss of BFA tolerance.

### Further exposure to Chr3x3 adaptors to higher amounts of brefeldin A selects aneuploid adaptors with diverse karyotypes

**Figure 6:**
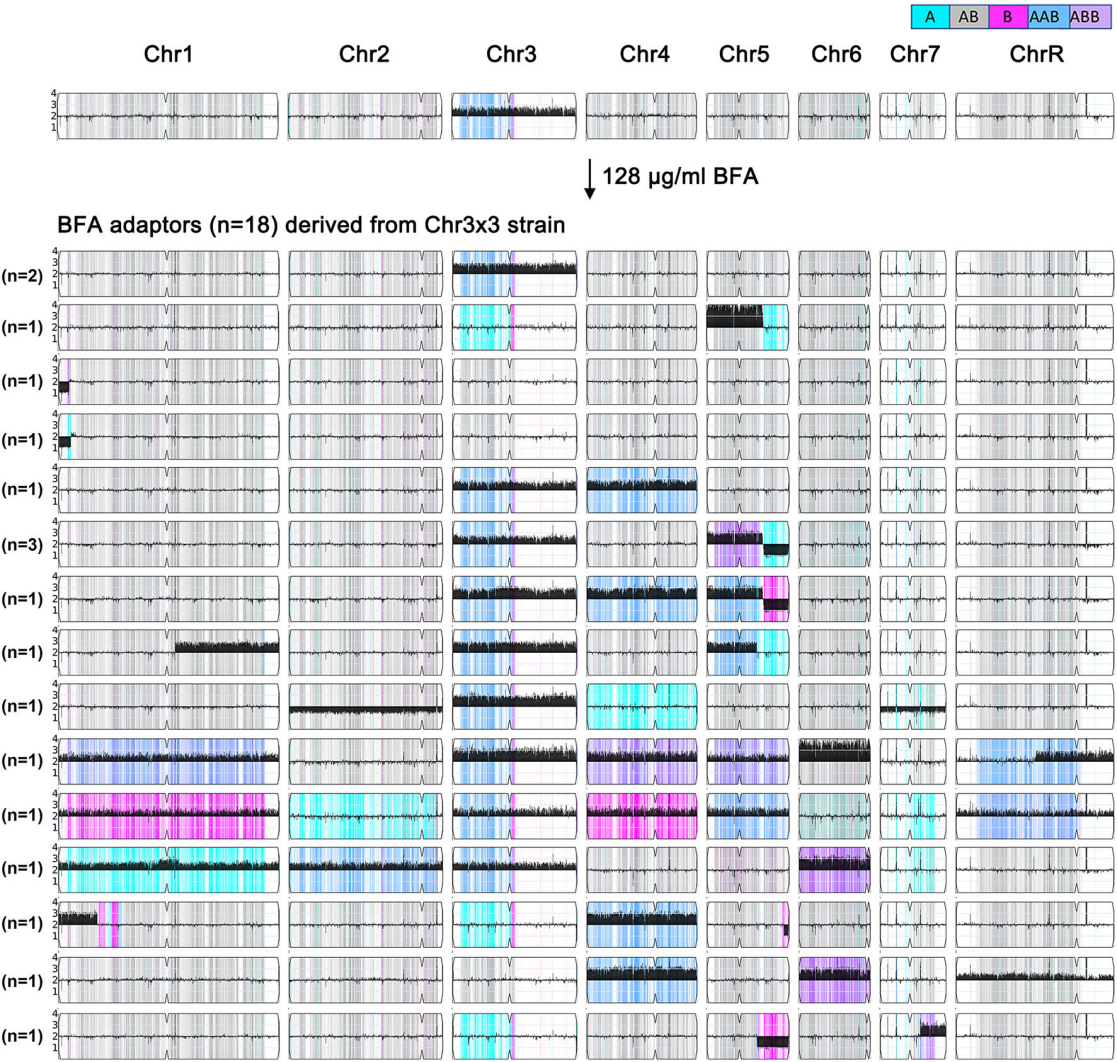
Chr3x3 drives genomic instability. One Chr3x3 strain (top) was spread on a YPD plate supplemented with 128 μg/mL of BFA. Eighteen adaptors were randomly selected and sequenced (bottom). These adaptors had 15 distinct karyotypes. The number of adaptors sharing the same karyotype is indicated on the left.

To further investigate how the Chr3x3 strain adapts to higher concentrations of BFA, No. 1—a Chr3x3 adaptor derived from SC5314 through exposure to 32 μg/mL BFA—was plated on YPD agar supplemented with 128 μg/mL BFA. From these, 18 adaptors were randomly selected (No. 1-1 to No. 1-18). Seventeen adaptors were more resistant than the parent to BFA (**Supplementary Figure S1**). All 18 adaptors were subjected to whole-genome sequencing. All 18 adaptors were aneuploid, exhibiting 15 distinct karyotypes. Among these, 12 adaptors retained Chr3x3, with 10 of these also gaining aneuploidy of additional chromosomes. The remaining six adaptors lost the original Chr3x3 but acquired aneuploidies of other chromosomes. Only five adaptors displayed aneuploidy of a single chromosome: two adaptors (No. 1–1 and No. 1-11) maintained only Chr3x3, one adaptor (No. 1-14) had tetrasomy of chromosome 5 (from the left telomere to 0.81 Mb), and two adaptors (No. 1–4 and No. 1-8) exhibited segmental monosomy of chromosome 1 (from the left telomere to 0.154 and 0.178 Mb, respectively). The other 13 adaptors carried aneuploidies of at least two chromosomes. For example, No. 1–12 had aneuploidies involving six chromosomes: trisomy of chromosomes 1, 3, 4, and 5; segmental trisomy of chromosome R; and tetrasomy of chromosome 6. Similarly, No. 1–7 displayed trisomy of five chromosomes (1, 3, 4, 7, and R), while No. 1–16 had trisomy of four chromosomes (1, 2, 3, and 6) (**Figure 6**). These findings demonstrate that a higher concentration of BFA facilitates the generation of increasingly diverse karyotypes.

**Figure 7:**
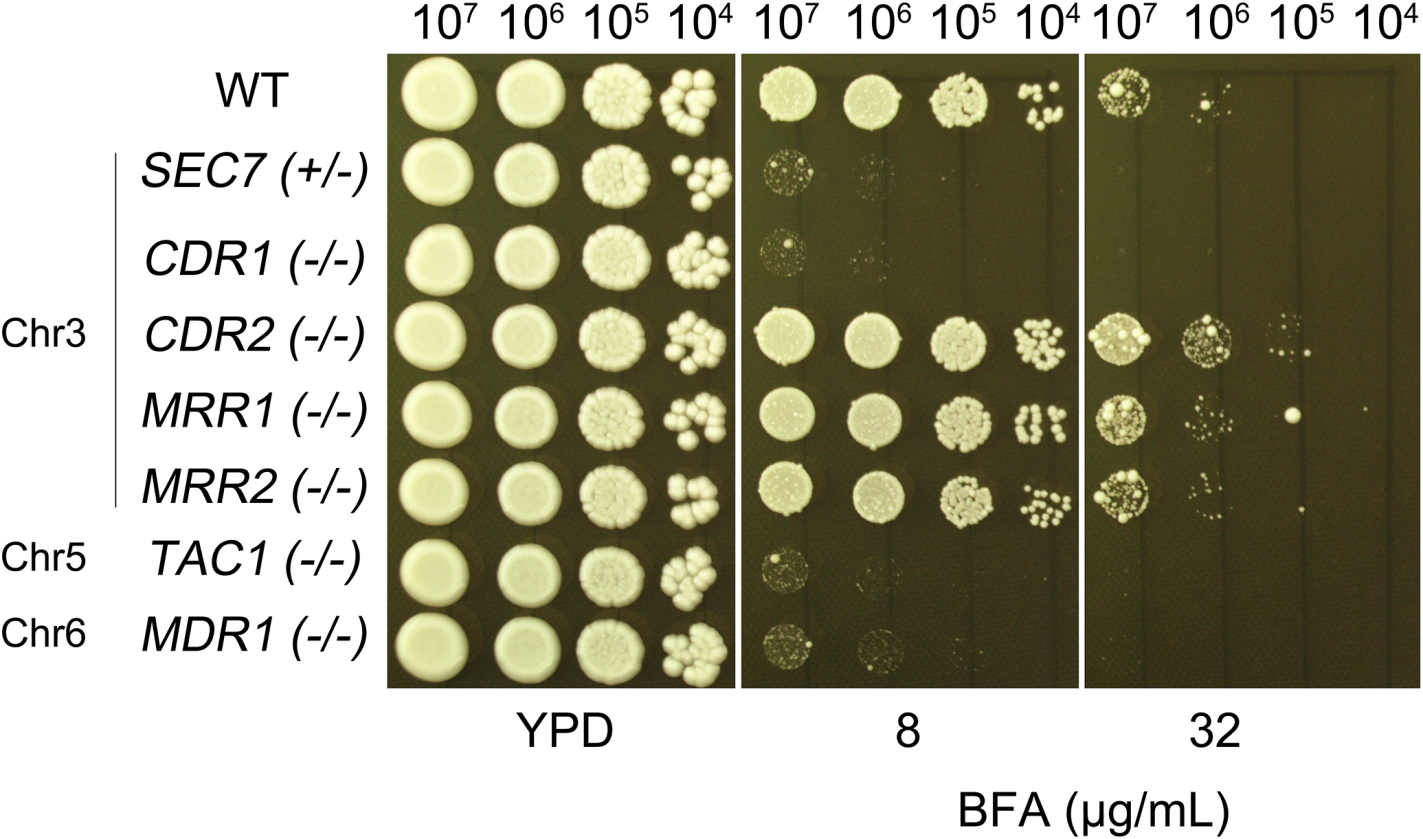
Chr5 and Chr6 genes are associated with BFA resistance. The wild-type control strain SC5314 and homozygous deletion mutants of *MRR1*, *MRR2*, *TAC1*, and *MDR1* were tested for resistance to BFA. Strains with homozygous deletions of *CDR1* and *CDR2*, as well as a heterozygous *SEC7* deletion, were included as controls. Tenfold serial dilutions of each strain were spotted onto YPD plates containing the indicated concentrations of BFA and incubated at 37°C for 48 h.

In addition to the previously identified Chr3 genes, *SEC7* and *CDR1*, which have been implicated in BFA resistance through gene dosage effects, we sought to determine whether other genes located on aneuploid chromosomes contribute to this phenotype. Given that aneuploidy can lead to broad transcriptional and functional changes, we hypothesized that additional resistance mechanisms might arise from altered copy numbers of key regulatory or efflux-related genes. To test this, we selected candidate genes based on their known or predicted roles in drug resistance: *MRR1* and *MRR2* (Chr3), *TAC1* (Chr5), and *MDR1* (Chr6). *MRR1* and *MRR2* encode transcription factors known to upregulate efflux pumps, while *TAC1* regulates the expression of multiple transporters involved in antifungal resistance. MDR1, on the other hand, encodes a major facilitator superfamily (MFS) transporter directly involved in multidrug efflux. Strikingly, deletion of *TAC1* or *MDR1* resulted in pronounced hypersensitivity to BFA, whereas loss of *MRR1* or *MRR2* had no significant effect (**Figure 7**). Notably, since *TAC1* is a transcriptional regulator, its deletion likely disrupts the expression of downstream efflux systems, whereas *MDR1* may mediate the direct extrusion of BFA or its toxic intermediates.

## Discussion

As a unicellular microorganism, *C. albicans* must rapidly and effectively adapt to environmental changes. Typically, cells respond to stress by transiently altering the expression of genes associated with stress adaptation (Estruch, 2000). However, *C. albicans* also employs an additional strategy: the formation of reversible aneuploidies. This pathogen exhibits remarkable genome plasticity, as evidenced by the diploid reference strain SC5314, which tolerates trisomy of any of its eight chromosomes (Yang et al., 2021b). Moreover, aneuploid strains are unstable in the absence of stress and spontaneously reverting to euploidy (Yang et al., 2021b). Under stress conditions—such as carbon source limitation, exposure to the echinocandin drug caspofungin, the chemotherapeutic agent hydroxyurea, or the ER stress inducer tunicamycin—specific aneuploidies rapidly arise, conferring tolerance to the particular stress. Upon removal of the stress, these aneuploid strains revert to euploidy, resulting in the concomitant loss of stress tolerance (Janbon et al., 1998; Yang et al., 2013; Yang et al., 2019; Yang et al., 2021a).

In this study, we found that exposure of the diploid wild-type strain SC5314 to both low and high concentrations of BFA was primarily selected for aneuploid adaptors. Among these, 77.8% (14 out of 18) carried aneuploidy of a single chromosome, predominantly trisomy of chromosome 3 (Chr3x3). Further exposure of the Chr3x3 adaptor to BFA yielded adaptors with increasingly diverse karyotypes, with 72.2% (13 out of 18) exhibiting aneuploidies of at least two chromosomes. These results demonstrate that aneuploidy serves as the primary mechanism for rapid adaptation to BFA in *C. albicans*, while also driving further genomic instability.

One advantage of developing aneuploidy is that it enables the simultaneous regulation of multiple genes, both on and off the aneuploid chromosome (Pavelka et al., 2010). In this study, we identified at least two genes on chromosome 3 associated with BFA tolerance: *SEC7* and *CDR1*. Both genes encode direct targets of BFA. These findings suggest that aneuploidy provides a rapid mechanism for coregulating the copy number of distinct genes that encode different targets of the same inhibitor, thereby facilitating adaptation to BFA stress.

Another advantage of aneuploidy is its reversibility. Adaptation to a specific stress often incurs fitness costs in the absence of that stress or in the presence of alternative stresses. These fitness trade-offs, also referred to as “costs of resistance”, are ubiquitous in nature and represent a fundamental principle in evolutionary biology (Wenger et al., 2011). Classical genetic mutations, such as point mutations and deletions, are irreversible. Relying on such mutations for adaptation to transient environmental changes can be risky for cells. In contrast, aneuploidy is reversible in pathogenic fungi, including *C. albicans* (Janbon et al., 1998; Yang et al., 2013; Yang et al., 2019; Yang et al., 2021a), *C. auris* (Bing et al., 2020), and *Cryptococcus neoformans* (Sionov et al., 2010; Chang et al., 2021). In the absence of stress, aneuploid strains spontaneously revert to euploidy (Tsai and Nelliat, 2019). In this study, we observed that aneuploidy enabled rapid adaptation to BFA. However, in the absence of BFA, all four aneuploid strains with distinct karyotypes reverted to euploidy and lost BFA tolerance. Thus, unlike the irreversible and potentially risky adaptation mechanism involving genetic mutations, aneuploidy offers a safe and reversible strategy for adapting to BFA stress.

Further exposure of the Chr3x3 strain to BFA resulted in the selection of adaptors with diverse aneuploidies. All adaptors were aneuploid, with 66.7% retaining Chr3x3 and 72.2% exhibiting aneuploidies involving at least two chromosomes. Notably, three out of 18 adaptors carried aneuploidies involving four to six chromosomes. These findings suggest that aneuploidy is maintained during subsequent exposure to the same stressor and may drive genomic instability in *C. albicans*. In cancer cells, aneuploidy is known to induce replication stress and promote genomic instability (Passerini et al., 2016). Similarly, in yeasts, aneuploidy has been linked to chromosome loss, defective mitotic recombination, impaired DNA damage repair (Sheltzer et al., 2011), and missegregation of even a single chromosome, which is sufficient to induce genome instability (Torres et al., 2008; Sheltzer et al., 2011; Zhu et al., 2012). We propose that Chr3x3 may drive genomic instability in *C. albicans* through analogous mechanisms.

In addition to Chr3x3, some BFA-tolerant adaptors exhibited other aneuploid karyotypes, including trisomy of chromosome 4 (Chr4x3), segmental tetrasomy of chromosome 5, segmental monosomy of chromosome 5, and segmental monosomy of chromosome 1. These findings suggest that genes located on other chromosomes or chromosomal regions may also contribute to BFA tolerance. Aneuploidy of these chromosomes or chromosomal segments could facilitate BFA tolerance by increasing the copy number of such genes. Alternatively, these aneuploidies might directly or indirectly regulate the expression or activity of *SEC7* and/or *CDR1*, further enhancing adaptation to BFA stress.

In conclusion, we demonstrated that trisomy of chromosome 3 (Chr3x3) enables reversible adaptation to both low and high concentrations of BFA in *C. albicans*. At least two genes on chromosome 3, *SEC7* and *CDR1*, were associated with BFA tolerance. Furthermore, Chr3x3 drove genomic instability during subsequent exposure to stronger selective pressure from the same stressor, leading to the emergence of adaptors with increasingly complex karyotypes. These findings highlight the dual role of aneuploidy as both a rapid adaptive mechanism and a driver of genomic instability in *C. albicans*.

## Data Availability

The datasets presented in this study can be found in online repositories. The names of the repository/repositories and accession number(s) can be found in the article/**Supplementary Material**.

## Supplementary material

Figure S1 Adaptive evolution of a Chr3x3 strain was performed by exposure to 128 μg/mL BFA. Eighteen randomly selected adaptors (#1-18) were subjected to spot assay analysis on YPD plates containing the indicated concentrations of BFA. The Chr3x3 progenitor strain (parent) was included as a control. All plates were incubated at 37°C for 48 hours before imaging.

Table S1 Sequences of primers used in this study.
