# Supplementary figures and images for: Aneuploidy enables adaptation to brefeldin A in Candida albicans
Source: Front Cell Infect Microbiol. 2025 Apr 28;15:1562726. doi: 10.3389/fcimb.2025.1562726 (PMC12066683; doi:10.3389/fcimb.2025.1562726)

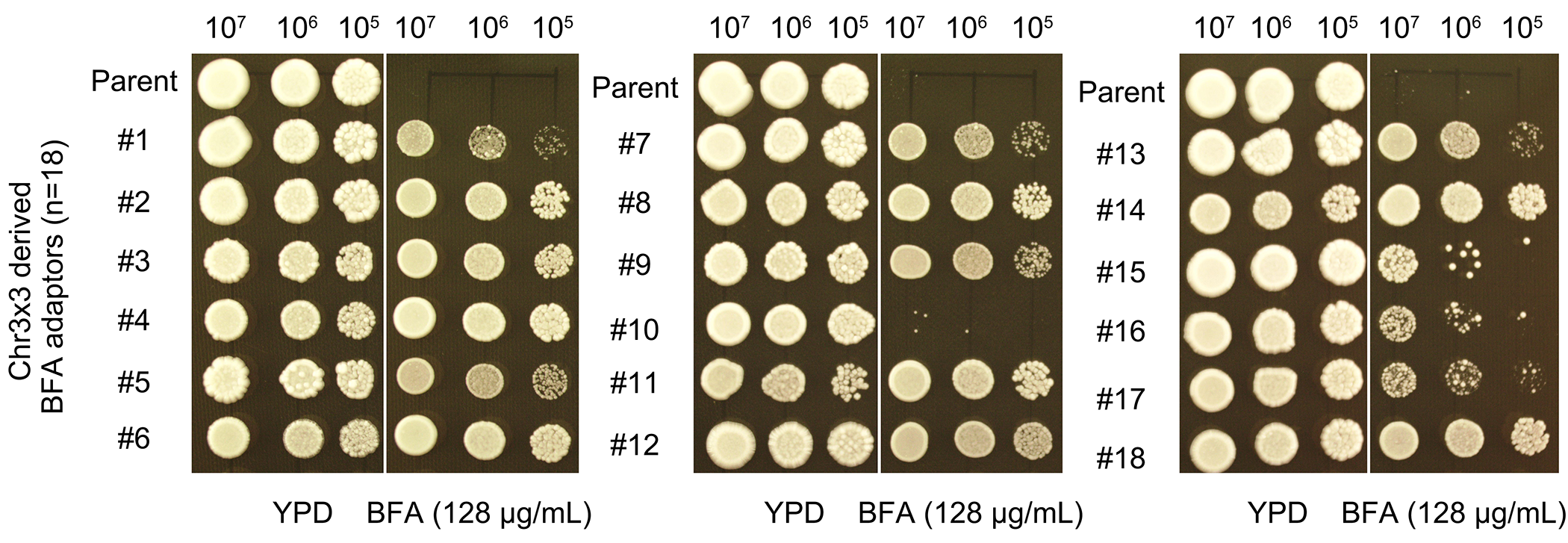

Supplement: Figure S1 — Adaptive evolution of a Chr3x3 strain was performed by exposure to 128 μg/mL BFA. Eighteen randomly selected adaptors (#1-18) were subjected to spot assay analysis on YPD plates containing the indicated concentrations of BFA. The Chr3x3 progenitor strain (parent) was included as a control. All plates were incubated at 37°C for 48 hours before imaging. [file Image1.tif]
