# Supplementary material for: Aneuploidy enables adaptation to brefeldin A in Candida albicans
Source: Front Cell Infect Microbiol. 2025 Apr 28;15:1562726. doi: 10.3389/fcimb.2025.1562726 (PMC12066683; doi:10.3389/fcimb.2025.1562726)
Supplement: Table S1 — Sequences of primers used in this study. [file Table1.docx]

Table S1. Sequences of primers used in this study

| Primer name | Primer sequence (5' to 3') |
| --- | --- |
| Gene deletions | |
| BP1639 | CAGATCGTACAATAAAGCTTTGAAG |
| BP1640 | TGCGTCTATTTATGTAGGATGAAAG |
| CaSEC7-US-F | CACTCGTGACGTTAATGGTG |
| NAT1-CaSEC7-US-R | GTATAGGAACTTCCTCGAGGGGGTTGTAGGCTGTGAGCTG |
| NAT1-CaSEC7-DS-F | AGATCCACTAGTTCTAGAGCGGCTGTAAGTGTATCATTTACCCAGC |
| CaSEC7-DS-R | CTGGTGTTTTCTTTCCTTTCC |
| CaCDR1-US-F | GGAGCTCAACGGAAAATTG |
| NAT1-CaCDR1-US-R | GTATAGGAACTTCCTCGAGGGCTTCTATTGGTGTGGGATCC |
| NAT1-CaCDR1-DS-F | AGATCCACTAGTTCTAGAGCGGGTAGACGTGGTGGTTAGTGGTC |
| CaCDR1-DS-R | CAGATGAGAAACACTTTTTCCC |
| CaCDR2-US-F | CCCTGTTGAAATTTCCACTC |
| NAT1-CaCDR2-US-R | GTATAGGAACTTCCTCGAGGGCAATCATTGTGGTATACATCGG |
| NAT1-CaCDR2-DS-F | AGATCCACTAGTTCTAGAGCGGCATTGGTAGCACCTTTCACC |
| CaCDR2-DS-R | CTGCCATGGTAATGATGTTG |
| CaMRR1-US-F | CTAATAAGGAAACTTTGCGGGAG |
| NAT1-CaMRR1-US-R | GTATAGGAACTTCCTCGAGGGGACATACAAAATCCAAACTTCGG |
| NAT1-CaMRR1-DS-F | AGATCCACTAGTTCTAGAGCGGGATTCTGGTGAATTGGCAGC |
| CaMRR1-DS-R | GCTTTTCGTATCACCAGATTGAG |
| CaMRR2-US-F | CGTGAACGATTGAGAGAGGTG |
| NAT1-CaMRR2-US-R | GTATAGGAACTTCCTCGAGGGGGGCAATTGCCTTGTCAC |
| NAT1-CaMRR2-DS-F | AGATCCACTAGTTCTAGAGCGGGCGTACCAAATCTTCCATGC |
| CaMRR2-DS-R | GATGTTTTATGGACGCAATGC |
| CaTAC1-US-F | GGGACGCAGTACATATAATAAAG |
| NAT1-CaTAC1-US-R | GTATAGGAACTTCCTCGAGGGGAGAACAACAGAATAGAGAGGG |
| NAT1-CaTAC1-DS-F | AGATCCACTAGTTCTAGAGCGGGAGTTGTAATTGGTGAAAGCG |
| CaTAC1-DS-R | GGGTATATGAATATAAAGTCTCGG |
| CaMDR1-US-F | GCTACCAATTAATCACAACGG |
| NAT1-CaMDR1-US-R | GTATAGGAACTTCCTCGAGGGGTTTGGTGTCTGATTCTTGC |
| NAT1-CaMDR1-DS-F | AGATCCACTAGTTCTAGAGCGGCGTGTTGCTTTTGCTTACG |
| CaMDR1-DS-R | GGCTAAGGTTATCCGTGTTC |
| Diagnostic PCR for deletions | |
| CaSEC7-USD-F | CTTGGTCAGCAAAGGAGG |
| CaSEC7-DSD-R | GCTCTCGTTCTGGGTAACTTC |
| CaCDR1-USD-F | GCACACACACACAAACACAC |
| CaCDR1-DSD-R | GACGTACATTGAAGGTTTGG |
| CaCDR2-USD-F | CACCTTTGTCTCCATATTCGTC |
| CaCDR2-DSD-R | CTTGGTGTTATTGGTGGTGG |
| CaMRR1-USD-F | GTGGGTTGTCAAATTTCCTGTC |
| CaMRR1-DSD-R | CGCTACCATAAGCCTCGC |
| CaMRR2-USD-F | CGACAGCTGGACCTTGG |
| CaMRR2-DSD-R | GTCGATCTGGGGCATTGAC |
| CaTAC1-USD-F | CCTTCTTTTAGCGCTTCC |
| CaTAC1-DSD-R | CCATAATGTATCCAATTTCCG |
| CaMDR1-USD-F | CGTTTAGTTGTTCCCAATCG |
| CaMDR1-DSD-R | GACACAAAACACGTACTTCGC |
